# Predictive investigation of idiopathic pulmonary fibrosis subtypes based on cellular senescence-related genes for disease treatment and management

**DOI:** 10.3389/fgene.2023.1157258

**Published:** 2023-03-24

**Authors:** Changqing Yang, Ziqi Han, Wenyu Zhan, Yubao Wang, Jing Feng

**Affiliations:** Respiratory Department, Tianjin Medical University General Hospital, Tianjin, China

**Keywords:** idiopathic pulmonary fibrosis, cellular senescence, subtype, prognosis, treatment

## Abstract

**Background:** Idiopathic pulmonary fibrosis (IPF), a chronic, progressive lung disease characterized by interstitial remodeling and tissue destruction, affects people worldwide and places a great burden on society. Cellular senescence is thought to be involved in the mechanisms and development of IPF. The aim of this study was to predictively investigate subtypes of IPF according to cellular senescence-related genes and their correlation with the outcome of patients with IPF, providing possible treatment and management options for disease control.

**Methods:** Gene expression profiles and follow-up data were obtained from the GEO database. Senescence-related genes were obtained from the CSGene database and analyzed their correlation with the outcome of IPF. A consensus cluster was constructed to classify the samples based on correlated genes. The GSVA and WGCNA packages in R were used to calculate the immune-related enriched fractions and construct gene expression modules, respectively. Metascape and the clusterProfiler package in R were used to enrich gene functions. The ConnectivityMap was used to probe suitable drugs for potential treatment.

**Results:** A total of 99 cellular senescence-related genes were associated with IPF prognosis. Patients with IPF were divided into two subtypes with significant prognostic differences. Subtype S2 was characterized by enhanced fibrotic progression and infection, leading to acute exacerbation of IPF and poor prognosis. Finally, five cellular senescence-related genes, TYMS, HJURP, UBE2C, BIRC5, and KIF2C, were identified as potential biomarkers in poor prognostic patients with IPF.

**Conclusion:** The study findings indicate that cellular senescence-related genes can be used to distinguish the prognosis of patients with IPF. Among them, five genes can be used as candidate biomarkers to predict patients with a poor prognostic subtype for which anti-fibrosis and anti-infection treatments could be suitable.

## 1 Introduction

Idiopathic pulmonary fibrosis (IPF) is a chronic, fibrotic interstitial pneumonia characterized by progressive hypoxemia and deterioration of lung function with overall poor prognosis (Raghu et al., 2022). IPF is thought to be caused by abnormal epithelial-fibroblast interaction induced by multiple factors, which results in deposition of extracellular matrix (ECM), and lung tissue reconstruction (Richeldi et al., 2017). It is estimated that an increasing global trend in the incidence of IPF occurred since 2000, with a high range of 3–9 cases per 100,000 people in some regions (Hutchinson et al., 2015), especially in North America, the incidence reached 7.5 to 9.3 per 100,000 people during the period of 2009–2019 (Maher et al., 2021). Additionally, in a U.S. study, the prevalence of IPF in elderly individuals over 65 years of age exceeded 400 cases per 100,000 in 2011 (Raghu et al., 2014). Similarly, the mortality of IPF is gradually increasing globally (Hutchinson et al., 2014), with a median survival time of only 3 years reported in the UK from 2000 to 2012 (Strongman et al., 2018) and 7.1 deaths per 100,000 reported in Australia in 2015 (Cox et al., 2022). A systemic review published in 2020 reported that the 5-year overall survival rates was only 31% (Khor et al., 2020). Among these, almost 40%–46% of deaths were associated with acute exacerbation of IPF (AE-IPF) (Jeon et al., 2006; Natsuizaka et al., 2014). However, a subset of patients with IPF have survived at least 5 years since diagnosis (Lederer and Martinez, 2018). Thus, specific biomarkers are needed to distinguish patients with IPF who would benefit from earlier intervention.

Cellular senescence, particularly alveolar epithelial cell senescence, is involved in the pathogenesis of IPF (Minagawa et al., 2011; Martinez et al., 2017). Alveolar epithelial type 2 cell (AEC2) senescence is generally considered to be a driver of IPF (Parimon et al., 2020). Cellular senescence refers to permanent cell cycle arrest in response to endogenous and exogenous stimuli, such as oncogene activation, mitochondrial dysfunction, and oxidative stress (Herranz and Gil, 2018). Senescent cells produce the senescence-associated secretory phenotype (SASP), such as cytokines, chemokines, and growth factors, that affect their microenvironment and create pro-inflammatory conditions (Coppe et al., 2008; Basisty et al., 2020; Di Micco et al., 2021). AEC2s experienced functional failure due to telomerase abnormalities that caused senescence, thus activating inflammatory responses and immune signals in a mouse model of IPF (Alder et al., 2015). In tumors, cGAS-STING (Gluck et al., 2017; Yang et al., 2017) which primarily regulates SASP in senescent cells, can induce an innate immune response and mediate immune surveillance (Dou et al., 2017). Similarly, senescent abnormal epithelial cells located at the edge of fibroblast foci may hinder the repair of epithelial cells and promote the fibrotic process (Adams et al., 2020). Senescence of alveolar type 2 (AT2) cells promoted progressive pulmonary fibrosis and led to poor prognosis in a novel mouse model of IPF (Yao et al., 2021). Additionally, telomere shortening, a mechanism that induces cellular senescence (Di Micco et al., 2021), is thought to be associated with overall and transplant-free survival in patients with IPF (Stuart et al., 2014; Planas-Cerezales et al., 2019). Therefore, we hypothesized that cellular senescence may affect the outcomes of IPF.

To investigate the relationship and effect of cellular senescence-related genes on IPF, in this predictive study, we used cellular senescence-related genes to identify subtypes of IPF distinguished by prognosis, to determine specific biomarkers for clinical prediction, and screened probable drugs for targeted treatment based on IPF subtype. The results provided a new perspective to improve the treatment and management of IPF.

## 2 Materials and methods

### 2.1 Data preparation and processing

The gene expression data, detected by microarray in bronchoalveolar lavage (BAL), and clinical data with survival-related information (Supplementary Table S1) of 176 patients with IPF extracted from the GSE70867 dataset were obtained from the Gene Expression Omnibus (GEO) database (https://www.ncbi.nlm.nih.gov/geo/query/acc.cgi?acc=GSE70867). All patients were diagnosed as IPF based on American Thoracic Society and European Respiratory Society criteria in a retrospective study (Prasse et al., 2019), regardless of sex and age. The BAL was examined for each patient before using treatments, such as pirfenidone and nintedanib. Death was recognized as the positive endpoint and censored as the negative endpoint in survival-related information. Data processing and analyses were performed using R version 4.1.3 and online tools. The dataset was mapped by GPL14550 and GPL17077 probes to get normalized gene expression values, and the batch effect was removed for subsequent analysis.

### 2.2 Identification of cellular senescence-related genes in IPF

A total of 503 cellular senescence-related genes were obtained from the CSgene database (http://csgene.bioinfo-minzhao.org/) (Zhao et al., 2016). After removing 40 genes that were not detected in the GSE dataset, 463 cellular senescence-related genes were identified for subsequent analysis. The karyoploteR package in R was used for genome display (Gel and Serra, 2017). The survival package in R was used to perform survival analysis, while survival time and status were calculated using the Cox proportional hazards regression model. Results with *p*-values <0.05 were selected for subsequent analysis. Kyoto Encyclopedia of Genes and Genomes (KEGG) pathway enrichment analysis was performed on significant genes using the clusterProfiler package in R (Wu et al., 2021), with *p*-values <0.01 considered to be significant. Transcriptional regulatory relationships unraveled by sentence-based text mining (TRRUST) enrichment analysis was carried out using Metascape (https://metascape.org/gp/index.html#/main/step1) (Zhou et al., 2019) to identify possible transcription factors regulating senescence-related genes.

### 2.3 Identification of cellular senescence-related subtype

Using the ConsensusClusterPlus package in R (Wilkerson and Hayes, 2010), a consistency matrix was constructed based on the expression of prognostic senescence-related genes. One hundred bootstraps were performed using the PAM algorithm and each bootstrap was guaranteed to involve 80% of the data in the original dataset. Clusters were set from 2 to 6. The t-distributed stochastic neighbor embedding (t-SNE) method was used to reduce the dimensionality of the data for display after classification. The survival information was used to calculate the median survival time using the Kaplan-Meier method, and the log-rank test was applied for significance comparison, with *p*-values <0.05 considered to be significant. Age and sex information was performed by the Wilcoxon and Chi-squared tests respectively, with *p*-values <0.05 as significant.

### 2.4 Cellular senescence-related subtype analysis

The enriched fractions of immune cells and pathways were analyzed *via* single-sample gene set enrichment analysis (ssGSEA) for the two subtypes using the GSVA package in R and published literature for immune cell types and pathways (Charoentong et al., 2017). The Wilcoxon rank-sum test was used to assess differences in immune cell infiltration and immune pathway enrichment between the two subtypes. The Limma package in R (Ritchie et al., 2015) was used to analyze differences in gene expression. *p*-values were corrected using the Benjamini–Hochberg method, and genes with adjusted *p*-values <0.05 and |log2FoldChange| >1 were considered to be differentially expressed genes (DEGs). Gene Ontology (GO) and KEGG analyses were conducted on the DEGs using Metascape. GO analysis included three aspects: biological processes, molecular functions, and cellular components. *p*-values <0.01 were considered to be significant pathways from the above enrichment analysis. By searching for similar expression signature caused by drug treatment, drug predictions for both subtypes were performed using the Connectivity Map (https://clue.io/) based on the DEGs (Subramanian et al., 2017).

### 2.5 Weighted gene co-expression network analysis

A total of 141 samples were included in the weighted gene co-expression network analysis (WGCNA) (Langfelder and Horvath, 2008, 2012). To determine the appropriate power value when independence was rounded to 0.9, an unsigned topology matrix was constructed, setting each module to contain a minimum of 30 genes and merging modules with a degree of dissimilarity <0.25. Combined with survival status and time, univariate Cox regression analysis was performed on the obtained gene modules. The prognostic module was fitted to the tsne1 component for a generalized linear model, hub genes which with a high degree of connectivity in the module were identified using the Molecular Complex Detection (MCODE) plugin (Bader and Hogue, 2003), and Cytoscape (Shannon et al., 2003) was used for visualization.

## 3 Results

### 3.1 Screening prognostic senescence-related genes in IPF

**FIGURE 1:**
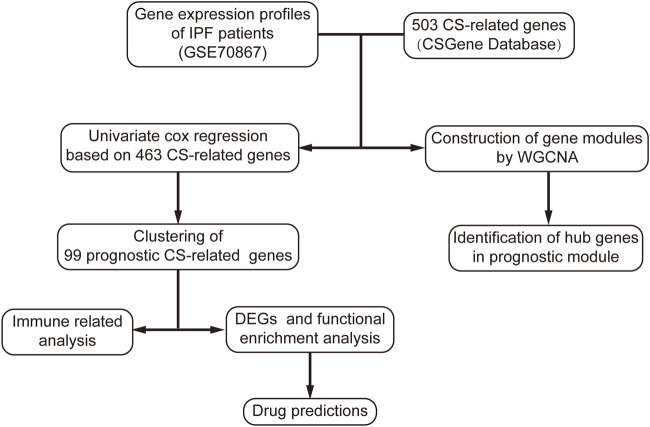
Flow chart of research process in this study. CS, cellular senescence.

**FIGURE 2:**
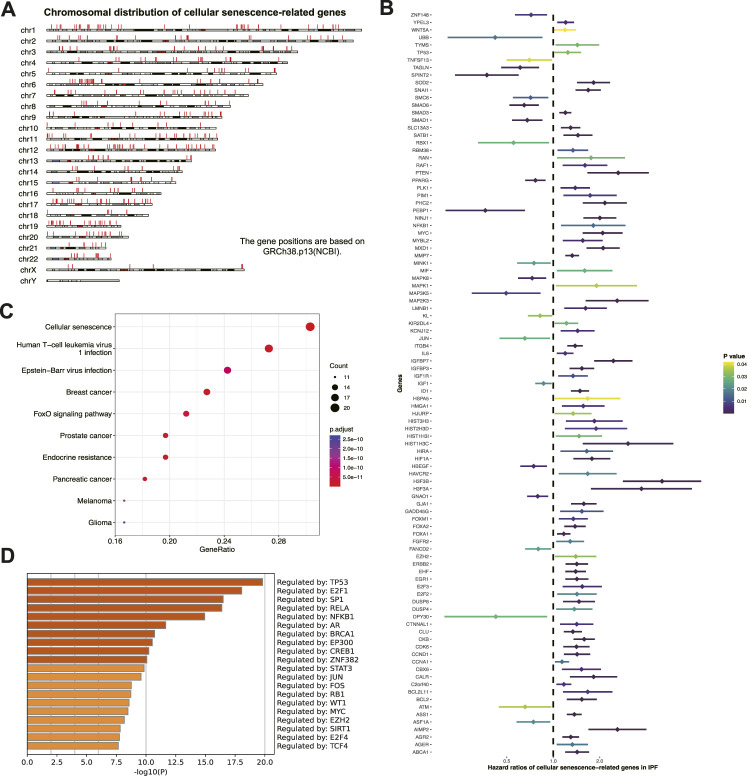
Screening prognostic cellular senescence-related genes in IPF. **(A)** Chromosomal distribution of cellular senescence-related genes in IPF. Each red line represents a gene. **(B)** Forest maps of single factor survival analysis of cellular senescence-related genes of IPF. **(C)** KEGG enrichment analysis of prognostic cellular senescence-related genes. Dot size corresponds to the count of genes in each pathway. **(D)** TRRUST enrichment analysis of prognostic cellular senescence-related genes.

The study workflow is shown in (Figure 1). Prognostic senescence-related genes were screened among 463 genes known to be associated with cellular senescence in the human genome (Figure 2A). Univariate Cox regression analysis was used to evaluate the above genes, and 99 prognostic senescence-related genes were identified (Figure 2B; Supplementary Table S2). The results indicated that genes such as SPINT2, PEBP1, and KL were associated with a protective effect against death, whereas genes such as HMGA1, ITGB4, and FGFR2 were risk factors of death in IPF. KEGG analysis showed the cellular senescence pathway was highly enriched for these genes (Figure 2C). Furthermore, the results suggested that transcription factors, such as TP53 and SP1, may have a regulatory effect on these genes and may contribute to the development of fibrosis (Figure 2D).

### 3.2 Identification of cellular senescence-related subtypes in IPF

**FIGURE 3:**
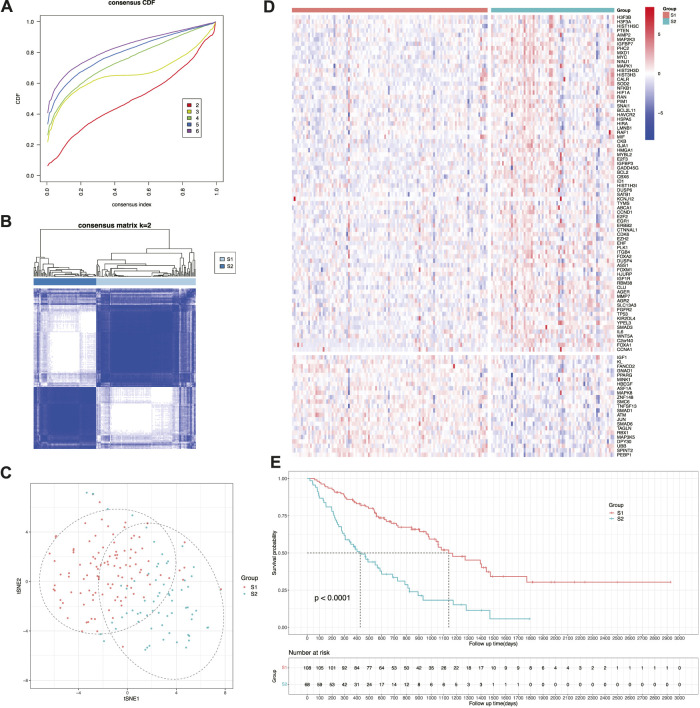
Identification of cellular senescence-related subtypes of IPF. **(A)** Cumulative distribution function curve. **(B)** Sample clustering heatmap. **(C)** t-SNE distribution of two subtypes. **(D)** Heatmap of two subtypes based on prognostic cellular senescence-related genes. **(E)** Kaplan-Meier curves showing overall survival of two subtypes.

To better manage the treatment of patients with IPF, samples were clustered into two prognostic subtypes designated as S1 and S2 based on the expression of the aforementioned genes (Figures 3A, B). Next, the dimensionality reduction method was used to construct the projection of the two subtypes in two-dimensional space. According to gene expression, samples were clearly divided into two groups, and the distinction in the first dimension of tSNE (tSNE-1) was relatively obvious (Figure 3C). By constructing a heatmap of gene expression values (Figure 3D), the expression of risk-related genes was relatively upregulated in subtype S2, whereas the expression of protection-related genes was relatively upregulated in subtype S1. Therefore, subtype S2 (median survival time: 426 days; 95% CI: 306–688 days) had a significantly shorter survival time than subtype S1 (median survival time: 1,140 days; 95% CI: 982–1769 days) (Figure 3E). Additionally, both subtypes had no significant difference in age (*p* = 0.455 > 0.05) and sex (*p* = 0.584 > 0.05). This indicated that the subtyping constructed by cellular senescence-related genes can effectively distinguish prognosis of patients with IPF, that is, subtype S2 has a worse prognosis than subtype S1.

### 3.3 Immune landscape of cellular senescence-related subtypes

**FIGURE 4:**
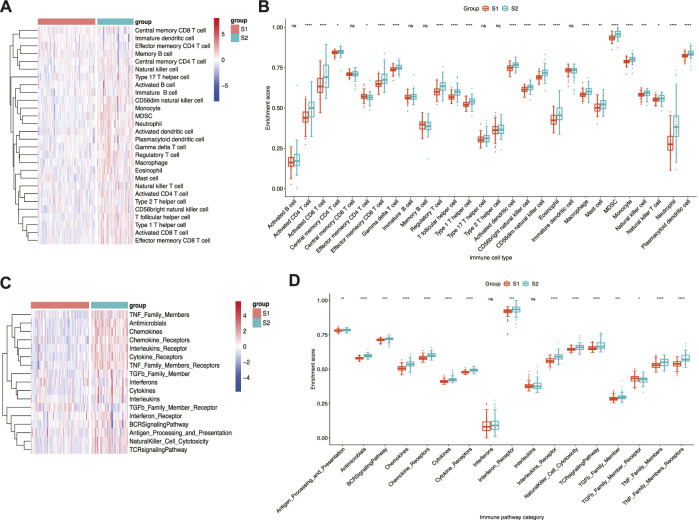
Immune landscape of senescence subtypes of IPF. **(A)** Heatmap and **(B)** boxplot of differential enrichment scores of immune cell signatures of two subtypes. **(C)** Heatmap and **(D)** boxplot of differential enrichment scores of immune pathway category of two subtypes. *p < 0.05, **p < 0.01, ***p < 0.001, ****p < 0.0001 and ns corresponds to no significance.

Given that pro-inflammatory cytokines are an important component of SASP (Basisty et al., 2020) and immune dysregulation contributes to IPF pathogenesis (Shenderov et al., 2021), the immune landscape of the two subtypes was analysed to determine whether immune-related factors differed between the two subtypes. The results indicated that immune cell infiltration was more pronounced in subtype S2 than in subtype S1 (Figure 4A). Moreover, greater infiltration of neutrophils and monocytes was observed in subtype S2 than in subtype S1, whereas Th2 and Th17 levels did not differ significantly between the two subtypes (Figure 4B). Similarly, subtype S2 displayed stronger immune pathway activation than subtype S1 (Figure 4C). Among these, antigen processing and presentation, antimicrobials, cytokines, chemokines, and TGF-β family members differed significantly between the two subtypes (Figure 4D). Overall, differences in immune cell infiltration and immune pathway enrichment were more pronounced in subtype S2, which may be related to poor prognosis.

### 3.4 Differentially expressed genes and drug probe of cellular senescence-related subtypes

**FIGURE 5:**
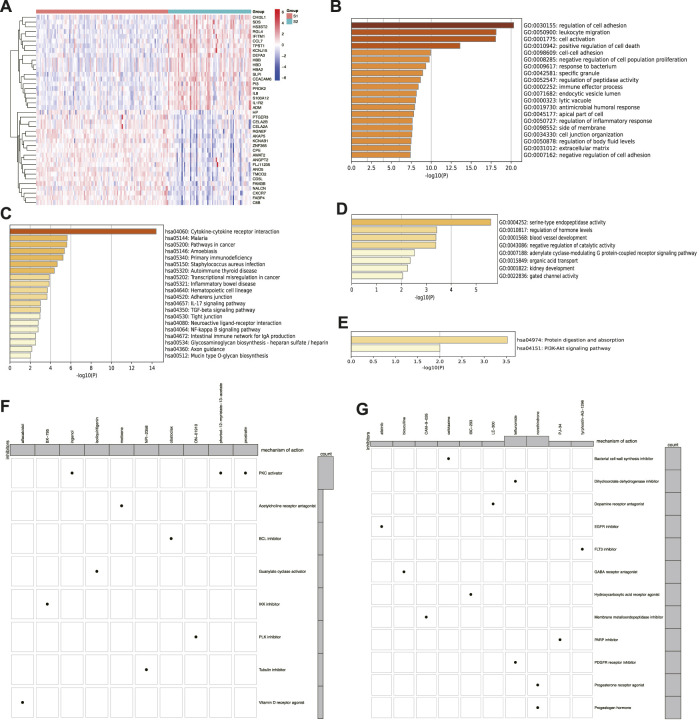
Differentially expressed genes and drug probe analysis of senescence subtypes of IPF. **(A)** Heatmap of top 20 up- and downregulated DEGs between two subtypes. **(B)** GO and **(C)** KEGG enrichment analysis of upregulated genes. **(D)** GO and **(E)** KEGG enrichment analysis of downregulated genes. **(F)** Subtype S1 and **(G)** subtype S2 drug probe and possible mechanism of each drug.

The DEGs (Supplementary Table S3) were comprehensively analyzed to better understand the differences between the two subtypes. The top 20 up- and downregulated DEGs in subtype S2 are shown in the heatmap (Figure 5A), demonstrating obvious differences between the two subtypes. Furthermore, GO and KEGG analyses were conducted on the up- and downregulated DEGs (Figures 5B–E). The upregulated DEGs were enriched in cell components and pathways, such as ECM, TGF-β, NF-κB, response to bacteria, leukocyte migration, and cytokine-cytokine receptor interaction. The downregulated DEGs were enriched in blood vessel development and the PI3K-Akt signaling pathway. According to the predicted functions of the DEGs, drugs such as leflunomide, tyrphostin-AG-1296, and PJ-34 may be effective against subtype S2, while ON-01910 (Rigosertib), Ingenol, and Isoliquiritigenin may be effective against subtype S1 (Figures 5F, G).

### 3.5 Identification of cellular senescence-related prognostic modules and hub genes

**FIGURE 6:**
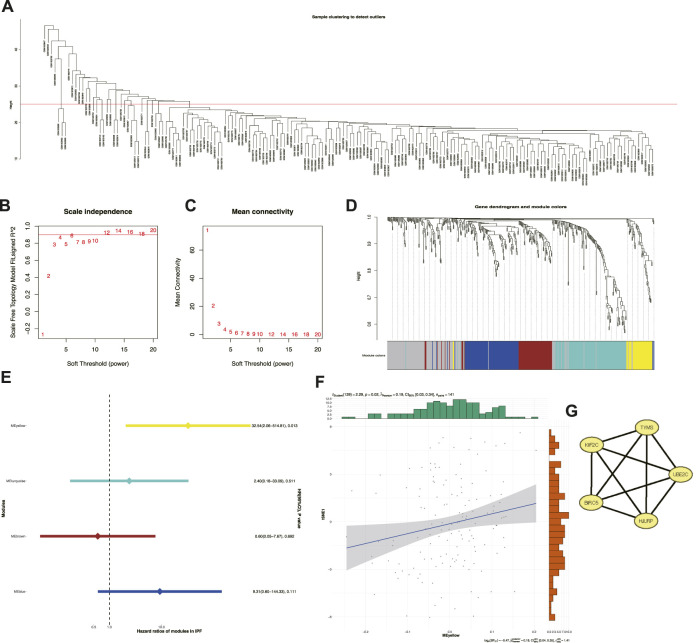
Identification of cellular senescence-related prognostic modules and hub genes. **(A)** Sample clustering. **(B)** Scale-free fit index for various soft-thresholding powers. **(C)** Mean connectivity for various soft-thresholding powers. **(D)** Dendrogram of cellular senescence-related genes clustered based on a one step method. **(E)** Forest maps of single factor survival analysis of modules of IPF. **(F)** Correlation between yellow module feature vector and second component of t-SNE. **(G)** Five hub genes identified in yellow module.

Key genes were explored among the 463 senescence-related genes to help clinicians distinguish patients with IPF with poor prognosis. Among them, a total of 35 outliers with obvious anomalies were excluded by the hierarchical clustering algorithm using the averaging method for 176 samples (Figure 6A). Taking the compliance and connectivity of the network into consideration (Figures 6B, C), four was chosen as the threshold to establish a scale-free network, and the topological matrix was obtained using a one-step method with four modules, shown in brown, blue, yellow, and turquoise (Figure 6D). Only the yellow module (Supplementary Table S4) had prognostic significance (HR: 32.54; 95% CI: 2.05-514.81). In addition, the yellow module was positively correlated with tSNE-1 (*r* = 0.19; *p* = 0.02; 95% CI: 0.03-0.34) (Figure 6E), suggesting that a higher gene fraction in this module indicated a greater likelihood of subtype S2. Given that the module score was closely related to the hub genes, gene-to-gene correlations >0.2 were identified as correlated genes and five hub genes were identified in the yellow module for subsequent studies (Figure 6F). Hence, TYMS, HJURP, UBE2C, BIRC5, and KIF2C were identified as potential biomarkers of subtype S2, for further prediction.

## 4 Discussion

Cellular senescence is implicated in the pathogenesis of IPF, and many studies have shown that it plays a pivotal role in the development of the disease. Most current research on senescence and IPF is based on experimental animal models. Senescence in epithelial cells, basal cells, and fibroblasts may be mechanically associated with the development of pulmonary fibrosis (Feng et al., 2019; DePianto et al., 2021; Yao et al., 2021). However, few studies have examined the relationship between cellular senescence and the prognosis of patients with IPF. To our knowledge, this is the first study to explore the relationship between cellular senescence-related genes and prognosis in IPF and to identify a cluster of patients with a significantly poor prognosis. The specific gene biomarkers identified in the study may help develop a risk assessment tool for patients with IPF, thereby facilitating easier judgement of prognosis and earlier intervention of IPF.

Cellular senescence is thought to have both protective and harmful effects on biological processes. This corresponds with the study findings in which prognostic senescence-related genes had a bilateral effect on patient outcomes. Gene regulation networks involve complex crosstalk, with each gene playing a particular role in the same biological process. For example, KL expression has been shown to exert a protective effect against overall mortality in non-diabetic, pre-dialysis chronic kidney disease (Yang et al., 2020), whereas FGFR2 expression was associated with increased risk of poor prognosis in patients with IPF and lung cancer (Li et al., 2018). Interestingly, two subtypes of IPF with different survival outcomes were identified based on the expression of these senescence-related genes. The study findings suggest that this classification system can distinguish the survival outcomes of patients with IPF for individualized management and treatment.

In the classification system applied in the present study, subtype S2 showed a prominent feature of IPF progression in which cellular senescence leads to increased fibrosis, resulting in poor prognosis. The deposition of ECM is an important process in fibrosis (Moss et al., 2022). TGF-β induces the transformation of fibroblasts into myofibroblasts, which promotes ECM production and collagen deposition (Fernandez and Eickelberg, 2012). ECM deposition can lead to tissue damage and immune cell infiltration, thereby promoting the fibrotic process. For example, neutrophils can activate fibroblasts by releasing extracellular traps (Chrysanthopoulou et al., 2014), while monocyte-derived macrophages, formed by recruited monocytes in the lungs, can also promote fibrosis (Misharin et al., 2017). Undoubtedly, these processes lead to the progression of fibrosis and possible development of AE-IPF (Collard et al., 2007). In addition, links between cellular senescence, NF-κB (Tian et al., 2019), and TGF-β (Kumari and Jat, 2021) have been previously demonstrated. Furthermore, NF-κB has been shown to promote the release of TGF-β (Wang et al., 2017). This suggests that cellular senescence can cause fibrotic progression through a series of downstream reactions, contributing to poor prognosis for subtype S2. However, subtype S2 also showed decreased gene enrichment in blood vessel development and the PI3K-Akt signaling pathway, which contradicts this speculation. Blood vessel development is an important component of interstitial lung disease (Ackermann et al., 2020) and the PI3K-Akt pathway contributes to pulmonary fibrosis (Sun et al., 2021). In the immune cell infiltration analysis, the abundance of Th2 and Th17 cells did not differ between the two subtypes. IL-4 and IL-13, produced by Th2 cells, can induce myofibroblast differentiation and trigger fibrotic effects (Hashimoto et al., 2001). Meanwhile, Th17 cells can produce IL-17, which activates fibroblasts and produces ECM (Zhang et al., 2019). In the immune pathway analysis, unlike the TGF-β family members that were highly enriched in subtype S2, TGF-β receptors were enriched in subtype S1. Taken together, these results partially support the hypothesis that cellular senescence can form a phenotype that promotes the progression of fibrosis, which in turn leads to poor prognosis. Nevertheless, available evidence supports this hypothesis. Indeed, pulmonary fibrosis was more severe in mice with AT2 cell senescence and the process of lung tissue fibrosis continued to deteriorate, leading to death (Yao et al., 2021). In addition, senescent cells can form a microenvironment of persistent pathological signaling in which SASP of senescent cells forms a pro-senescence loop by activating p38 MAPK and JAK, which in turn induces fibroblast senescence and mediates the continuous deposition of ECM (Rackow et al., 2020). Thus, this process contributes to the progression of pulmonary fibrosis.

The study findings also support another hypothesis, in that cellular senescence may lead to defects in epithelial cell function, causing impaired barrier function and susceptibility to infection that can lead to progression or even death in patients with IPF. Patients with a high proportion of neutrophils in BAL reportedly had a higher risk of early mortality (Kinder et al., 2008). In addition, patients with high monocyte count levels in peripheral blood had a higher risk of mortality and hospitalization (Kreuter et al., 2021). When pathogenic microorganisms invade the lungs, the mononuclear macrophage system is activated, inducing a powerful pro-inflammatory response (Albright et al., 2016). The process of clearing the pathogenic microorganisms may cause secondary injury to lung tissue that has already developed structural abnormalities, leading to further deterioration. In addition, given that IPF is more common in the elderly (Schneider et al., 2021), we suspect that this immune response to infection in subtype S2 may aggravate patients with IPF who are already in poor condition, resulting in AE-IPF triggered by infection (Invernizzi and Molyneaux, 2019). Previous studies have shown that *Streptococcus* and *Staphylococcus aureus* can be detected in the BAL of patients with AE-IPF in contrast to those in stable condition. This microbiome change is associated with disease progression (Han et al., 2014) and bacterial burden is closely related to IPF progression and increased mortality (Molyneaux et al., 2014). Unlike bacteria, viral infections do not appear to be related to the development of AE-IPF. Although torque tenovirus is highly detected in the BAL of patients with AE-IPF, the virus is not clearly related to disease progression (Wootton et al., 2011). Interestingly, studies report that epithelial cell dysfunction caused by cellular senescence may lead to increased bacterial and viral infections (Metzger and Sun, 2013; Torrance and Haynes, 2022). These findings concur with our hypothesis that pathogenic microbial infections, especially by bacteria, can easily occur in subtype S2, causing AE-IPF and poor prognosis.

Furthermore, the present study provides insight into drug screening for both subtypes. Leflunomide is a common drug used as a background therapy for rheumatoid arthritis, and another study reported that it can attenuate fibrosis in systemic sclerosis (Morin et al., 2017). Although PJ-34, a PARP inhibitor, has not been directly applied to the study of fibrotic diseases, studies have described the role of PARP inhibitors in fibrosis and their potential effectiveness against IPF (Rao et al., 2020). Interestingly, cefotaxime, a third-generation cephalosporin antimicrobial, was among the suitable drugs identified for subtype S2, which aligns with our hypothesis regarding pathogenic microbial infections easily occurring in subtype S2 and leading to poor prognosis. Ingenol can weaken fibroblast growth in keloids by promoting apoptosis (De Felice et al., 2018). Isoliquiritigenin may improve tubular fibrosis in diabetic nephropathy (Lin et al., 2022). The study findings indicate that the application of anti-fibrosis and anti-infection drugs may delay the progression of fibrosis and curb the development of AE-IPF. Thus, these potential drugs may be effective against IPF, especially subtype S2.

Five genes were identified to clinically predict subtype S2, including TYMS, HJURP, UBE2C, BIRC5, and KIF2C. Among them, TYMS, KIF2C, UBE2C, and HJURP are associated with unfavorable prognosis in hepatocellular carcinoma, possibly due to the malignant growth of cells (Ieta et al., 2007; Chen et al., 2018; Wei et al., 2021; Wang et al., 2022). TYMS, which encodes thymidylate synthetase, is involved in DNA replication and repair. IPF is characterized by fibroblast proliferation and differentiation, suggesting that elevated TYMS expression in IPF may indicate an ongoing fibrotic process. In addition, DNA damage can cause cellular senescence, suggesting that high TYMS expression may herald DNA repair in senescent cells. KIF2C, UBE2C, and HJURP are associated with mitotic processes and regulation of the cell cycle (Rape and Kirschner, 2004; Moore et al., 2005; Dunleavy et al., 2009). High expression of these genes may lead to prolonged cell cycle entry into cellular senescence and continued secretion of pro-fibrotic cytokines, resulting in progression of IPF. BIRC5 is associated with early activation of hepatic astrocytes and the development of hepatic fibrosis (Sharma et al., 2021). Interestingly, BIRC5 encodes an apoptotic inhibitory protein that regulates the immune system and inflammatory response (Sharma et al., 2017). Concurring with our hypotheses, the expression of BIRC5 can also lead to the progression of fibrosis or the susceptibility to infection. Therefore, screening for these genes can be used as a more economical and rapid test in the clinical setting. However, convenient tests usually lose accuracy, which may result in ambiguous prediction. As for this situation, detection of 99 prognostic senescence-related genes can be used to properly judge the subtypes. In summary, these genes are likely to be significantly related to the progression of fibrosis and poor prognosis through cellular senescence, thereby providing potential biomarkers for subtype S2 prediction.

This study had some limitations. The lack of survival information data for patients with IPF prevented finding a validation cohort to confirm the reliability of the two subtypes. In addition, the study findings do not confirm whether cellular senescence produces considerable pro-fibrotic effects *in vivo* or elucidate the underlying molecular mechanisms. Further, poor prognosis due to infection-induced progression of fibrosis does not exclude other causes of death induced by the infection itself, such as acute respiratory distress syndrome. Moreover, the specific effects of the identified drugs remain to be experimentally confirmed. Therefore, *in vitro* and *in vivo* experiments will be performed in the future to verify and evaluate the results of the present study in terms of immune cell infiltration, immune pathways, and candidate drugs. Clinical trials are also needed to determine the importance of infection in the adverse outcomes of patients with IPF. Similarly, the diagnostic genes for subtype S2 should be evaluated in large clinical samples.

In summary, this predictive study provides new insights into the etiology, treatment, and management of patients with IPF. For such patients with potential rapid progression and poor prognosis based on the classification system described herein, clinicians should intervene early, paying greater attention to general conditions, clinical symptoms and signs, and objective examination indicators, offering assistance in standardized management and drug therapy as appropriate during clinical follow-up. For patients with a long-lasting dry cough and a suspicious, new ground-glass shadow on CT scans, clinicians should be vigilant about the progression of IPF and intervene with treatment at an early stage.

## 5 Conclusion

This predictive study demonstrates a cellular senescence-based IPF subtyping approach that distinguished patients with poor prognosis and identifies anti-fibrosis and anti-infection agents as possible options for targeted treatment and management. TYMS, HJURP, UBE2C, BIRC5, and KIF2C were identified as potential biomarkers for poor prognostic patients with IPF.

## Data Availability

The original contributions presented in the study are included in the article/Supplementary Material, further inquiries can be directed to the corresponding authors.

## Supplementary material

SUPPLEMENTARY TABLE S1 Clinical information. The clinical information of patients included in this study.

SUPPLEMENTARY TABLE S2 99 prognostic CS-related genes. A total of 99 prognostic cellular senescence-related genes obtained from the univariate Cox regression analysis.

SUPPLEMENTARY TABLE S3 Differentially Expressed Genes. Differentially expressed genes obtained from the differential analysis.

SUPPLEMENTARY TABLE S4 Genes in yellow module. A total of 44 genes in yellow module obtained from the weighted gene co-expression network analysis.
